# Herd-level bovine tuberculosis risk factors: assessing the role of low-level badger population disturbance

**DOI:** 10.1038/srep13062

**Published:** 2015-08-17

**Authors:** David M. Wright, Neil Reid, W. Ian Montgomery, Adrian R. Allen, Robin A. Skuce, Rowland R. Kao

**Affiliations:** 1 School of Biological Sciences, Queen’s University Belfast, Belfast, BT9 7BL, Northern Ireland; 2 College of Medical, Veterinary and Life Sciences, University of Glasgow, Glasgow, G61 1QH, Scotland; 3 Quercus, School of Biological Sciences, Queen’s University Belfast, Belfast, BT9 7BL, Northern Ireland (UK); 4 Institute for Global Food Security (IGFS), Queen’s University Belfast, Belfast, BT9 5BN, Northern Ireland (UK); 5 Veterinary Sciences Division, Agri-Food and Biosciences Institute, Stormont, Belfast BT4 3SD, Northern Ireland

## Abstract

Bovine TB (bTB) is endemic in Irish cattle and has eluded eradication despite considerable expenditure, amid debate over the relative roles of badgers and cattle in disease transmission. Using a comprehensive dataset from Northern Ireland (>10,000 km^2^; 29,513 cattle herds), we investigated interactions between host populations in one of the first large-scale risk factor analyses for new herd breakdowns to combine data on both species. Cattle risk factors (movements, international imports, bTB history, neighbours with bTB) were more strongly associated with herd risk than area-level measures of badger social group density, habitat suitability or persecution (sett disturbance). Highest risks were in areas of high badger social group density *and* high rates of persecution, potentially representing both responsive persecution of badgers in high cattle risk areas and effects of persecution on cattle bTB risk through badger social group disruption. Average badger persecution was associated with reduced cattle bTB risk (compared with high persecution areas), so persecution may contribute towards sustaining bTB hotspots; findings with important implications for existing and planned disease control programmes.

Bovine tuberculosis (bTB), caused by the bacterium *Mycobacterium bovis*, has proven difficult to control and eradicate in Irish cattle, despite most EU countries being officially disease free. Intensive ongoing efforts to control the disease are based on a Government sponsored regime of tuberculin testing and slaughter of infected cattle, an expensive programme for both farmers and the government^1^. A range of herd-level risk factors for bTB have been identified in previous studies, although the composition of such lists varies depending on the context, scale of investigation and study period^2^,^3^,^4^,^5^.

The presence of a wildlife reservoir of infection (the Eurasian badger *Meles meles*) has been identified as a major factor contributing towards the difficulty of bTB eradication in Ireland^6^. However, the importance of badgers in maintaining the cattle epidemic is extremely controversial^7^,^8^,^9^, and the ways in which badger population dynamics affect levels of bTB in cattle populations at national scales are little understood with few large-scale studies^10^,^11^. In parts of Great Britain where badger populations have increased rapidly (i.e. south-west England)^12^,^13^, the estimated risk of bTB infection at the herd-level is positively associated with badger population density^10^. However, the absence of associations between cattle bTB risk and badger population densities in other areas may simply be due to low prevalence of bTB infection in both the cattle and badger populations such that there is an insufficient signal to noise ratio for an association to be detected.

The effect of badger population disruption has been debated in the context of specific culling efforts to control bTB. Whilst large-scale proactive culling of badgers has been associated with decreased bTB inside cull areas^9^,^14^, culling of badgers at both large and small spatial scales has been associated with increased bTB incidence in herds neighbouring cull areas with the mechanism thought to be badger social group disruption and migration (so called ‘perturbation’)^9^,^15^,^16^,^17^,^18^. Besides officially sanctioned culls there is evidence of low but sustained levels of illegal culling and sett disturbance (badger persecution) across Great Britain^12^,^19^,^20^.

Here we investigated, for the first time, whether there is evidence for an association between cattle bTB risk and illegal persecution of badgers. We used a spatially explicit model to assess not only the potential contribution of badger social group density to the dynamics of bTB but also its interaction with persecution (sett interference). The perturbation hypothesis states that when a badger sett is disturbed or some resident badgers killed, surviving social group members are more likely to disperse, increasing contact rates with badgers in neighbouring social groups and potentially spreading bTB^16^. However, while in areas with high levels of persecution we might expect to find increased incidence of bTB, the impact of low levels are as yet unquantified.

We combined data on the cattle and badger populations in Northern Ireland to produce a comprehensive list of risk factors concentrating on aspects of bTB risk potentially influenced by interactions between the two host populations. We also addressed several other important issues including the impact of international, cross-border cattle imports. By identifying herd-level risk factors of bTB infection, we aim to aid the eradication programme by informing farmers, veterinarians and policy-makers on the strategies most likely to reduce disease incidence. We specifically address the following questions: i) What are the cattle-related risk factors for herd-level bTB detection (herd breakdowns)? ii) Is cattle bTB risk associated with badger social group density and/or habitat suitability? iii) Is badger persecution (specifically, sett interference) associated with increased bTB risk?

## Methods

### Bovine tuberculosis in cattle

Anonymised tuberculosis test results (tuberculin skin test) and animal movement data for all active cattle herds (i.e. those with animals present at the beginning of the calendar year) in Northern Ireland during the period 2004 to 2011 were made available from the Animal and Public Health Information System (APHIS^21^) curated by the Department of Agriculture and Rural Development (DARD). We calculated eight variables describing potential bTB risk for each of 185,589 farm-years spread among 29,513 cattle herds (Table 1).

### Badger population data

Data describing the badger population were provided courtesy of DARD from the ‘Badger Survey of Northern Ireland 2007/08’^22^,^23^. For details of access arrangements for both datasets, please contact the corresponding author. The most south-westerly 1 km square in each 10 km square in Northern Ireland was surveyed for badger activity, along with additional 1 km squares in areas expected to have high badger density (total *n* = 212, Supplementary materials Fig. 1). The number of main setts (also taken as the number of badger social groups) was surveyed in each square (for full methodology of sett classification see^22^). Badger population variables were stored as layers within a Geographical Information System (GIS). We extracted two measures of badger abundance for each herd location (Table 1); mean social group density (i.e. active main setts) per 1 km^2^ (interpolated between survey sites using the Kriging tool in Spatial Analyst for ArcGIS 10.2; ESRI, California, USA) and an index of habitat suitability derived from a spatially explicit Species Distribution Model (SDM) associating badger main sett presence with landscape features (for full methodology of sett classification see^22^). An index of badger persecution (range 0–1) was also extracted by Spatial Kriging, indicating the probability of interference with sett structure during 2007/08 recorded as (1) recent digging, (2) entrances being blocked with soil, boulders, branches or other debris inserted directly into holes, (3) dumping of farm debris including bricks on top of setts, (4) agricultural disturbance such as setts being ploughed over or damaged by livestock trampling, (5) development such as the construction of roads or newly built houses and (6) other sources of disturbance such as slurry being pumped into holes^20^. Values were extracted for each cattle herd location, represented by the point location of the main farmhouse/buildings. Farms in Northern Ireland tend to be small (modal farm size of 20–30 ha^24^) and so attributes extracted at the farm building location are likely to be representative of the overall farm characteristics.

### Statistical analysis

Herd-level incidence of new bTB breakdowns was examined using logistic regression. Incidence was defined as the number of new breakdowns in a given year divided by the number of herds active during that year. A herd scored 1 if there was a new confirmed breakdown (defined by positive laboratory culture of *M. bovis*) within a given year and 0 otherwise (in Northern Ireland all herds are tested at least once a year, in comparison with Great Britain where testing frequency varies geographically with bTB risk). Herds with unconfirmed or inconclusive skin test results scored 0. The aim was to identify potential risk factors associated with new herd breakdowns so herds undergoing a continuing breakdown were excluded from the risk-set (denominator of the incidence calculation) until the calendar year following that in which the breakdown had been declared over, which occurs after two subsequent negative follow-up tests occurring at least 60 days apart (i.e. a breakdown extending over two years would only be counted in the first year). A summary of the number of herds in each category is given in supplementary Table S1. When a breakdown occurs neighbouring herds are then tested (lateral check tests); breakdowns detected as a result of these were also included in the analysis as we wanted to examine the effect of test results for neighbouring herds. In this analysis, neighbouring herds were defined as having locations (of main farm buildings) within a 1 km radius of the focal farm, a distance chosen because the average number of neighbours within this radius (8.75) was similar to survey-based estimates of the average number of neighbouring farms with contiguous boundaries to a focal farm^25^. Candidate explanatory variables were screened prior to multivariable modelling and only those associated with bTB incidence at *P* < 0.1 in univariable models were retained (Table 1). Year was fitted as a random factor to account for temporal trends in incidence and a separate random effect was also fitted for each herd to adjust for residual farm-level dependencies in risk (e.g. some business practices might increase risk in a manner not captured by the measured cattle variables). Badger population variables were standardised, subtracting the mean and scaling by 2 standard deviations (SD). This transformation allows approximate comparison of effect sizes with those of binary variables because the difference between outcomes for binary variables is approximately 2 SD and so coefficients for the rescaled continuous variables will represent a similar difference. Variables describing herd size and number of cattle movements were categorised into bands broadly representative of different farm business models (smallholder, medium-sized traditional farm, large-scale intensive farm; closed vs. open herd) for comparability with previous studies^10^. A base cattle-only model was fitted and compared with models with various combinations of cattle and badger population variables; better fitting models were selected as having lower Akaike’s Information Criterion (AIC values). Models assuming different functional forms of the relationship between badger variables and bTB risk (i.e. transformed predictors) were also compared with those assuming linear relationships. As a sensitivity analysis the selected cattle-badger model was refitted using only data from herds located in grid squares in which badger setts were surveyed, to determine whether patterns observed using the full dataset were consistent with those where badger variables were measured (rather than spatially interpolated).

Models were fitted using the *lme4* package in *R* version 3.0^26^,^27^ and assessed using the Area Under the Curve (AUC value) derived from the Receiver Operating Characteristic (ROC) curve. The effect of residual spatial variation was assessed by inspecting variograms for each year up to a maximum distance of 15 km using the *geoR*^28^ package (Supplementary Fig. 2).

### Temporal trends in bTB risk

To further investigate relationships among cattle and badger variables, we conducted a descriptive analysis comparing temporal trends in bTB incidence in cattle in areas with high and low badger persecution, both before and after the badger population data were collected (autumn-winter 2007/08) in order to infer the directionality of any identified relationships between persecution and bTB incidence. High incidence in areas subsequently found to have relatively high persecution would be consistent with persecution being largely a response to high local bTB risk. We also reasoned that if persecution reduced risk we might expect a disproportionately large decrease in incidence immediately after the badger survey in areas with high persecution compared with low persecution areas, over and above any longer term incidence trends. However, such an effect might be obscured if patterns of persecution changed dramatically in the period immediately after the badger survey (e.g. if persecution increased in low persecution areas and *vice versa*), so changes in incidence cannot be unambiguously associated with persecution at a particular time point. The study period was split into two year intervals (2004/05, 2006/07, 2008/09 and 2010/11) and the selected cattle-badger model was fitted to data from each interval separately. Herd-level predictions of bTB risk were extracted and used to calculate expected bTB incidence by badger main sett density. Preliminary analysis showed persecution risk was low but had a strongly right-skewed distribution. Herds in areas with persecution indices in the upper quartile of the distribution (probability of sett disturbance >0.35) were perceived as high persecution areas representing 29% (2,941 km^2^) of the land area with active cattle herds compared with herds in other areas (i.e. perceived low persecution over 7,355 km^2^).

## Results

There was a total of 8,864 new cattle herd bTB breakdowns in our dataset from 2004 to 2011 (Supplementary Table S1) Incidence of new herd breakdowns declined by 62% during the same period from 6.3% to 2.4%. There was substantial support for the model containing both cattle and badger population variables (Table 2, AIC values: cattle only model = 64,405; badger only model = 69,842; combined model = 64,106) though predictive power was modest (AUC = 0.763). Predictive power was higher for the badger only model but this is likely to have been due to over-fitting (see discussion in supplementary materials). There was no evidence that the relationships between bTB risk and badger variables were non-linear (non-linear specifications did not substantially improve model fit, Supplementary materials - Table S3). New breakdown herds had marked differences in characteristics to non-breakdown herds (Supplementary Table 2). Herd size was associated with the largest variation in risk of bTB breakdown (more than two-fold increase in risk for large relative to medium-sized herds, Table 2). There was no difference in risk between beef and dairy herds. Risk of breakdown was elevated by more than two-fold in herds that had experienced a breakdown during the previous year relative to herds with no breakdowns during the past decade. This effect was attenuated as time since previous breakdown increased but herds with breakdowns 6–10 years previous remained at 44% increased risk. There was a positive association between risk of breakdown and whether neighbouring farms experienced a bTB breakdown during the previous year (risk increased by 26% for each infected neighbouring farm) and a very weak negative association with the total number of neighbours. Risk also increased with the number of cattle movements; importing up to 10 batches of cattle from herds within Northern Ireland increased the risk by a third whilst importing more than 10 batches of cattle more than doubled the risk of a breakdown. Moreover, importing animals from the Republic of Ireland was associated with an additional increase in risk of 17% (Table 2). Associations between bTB risk and cattle variables were consistent throughout the study period (supplementary material, Table S4).

**Figure 1:**
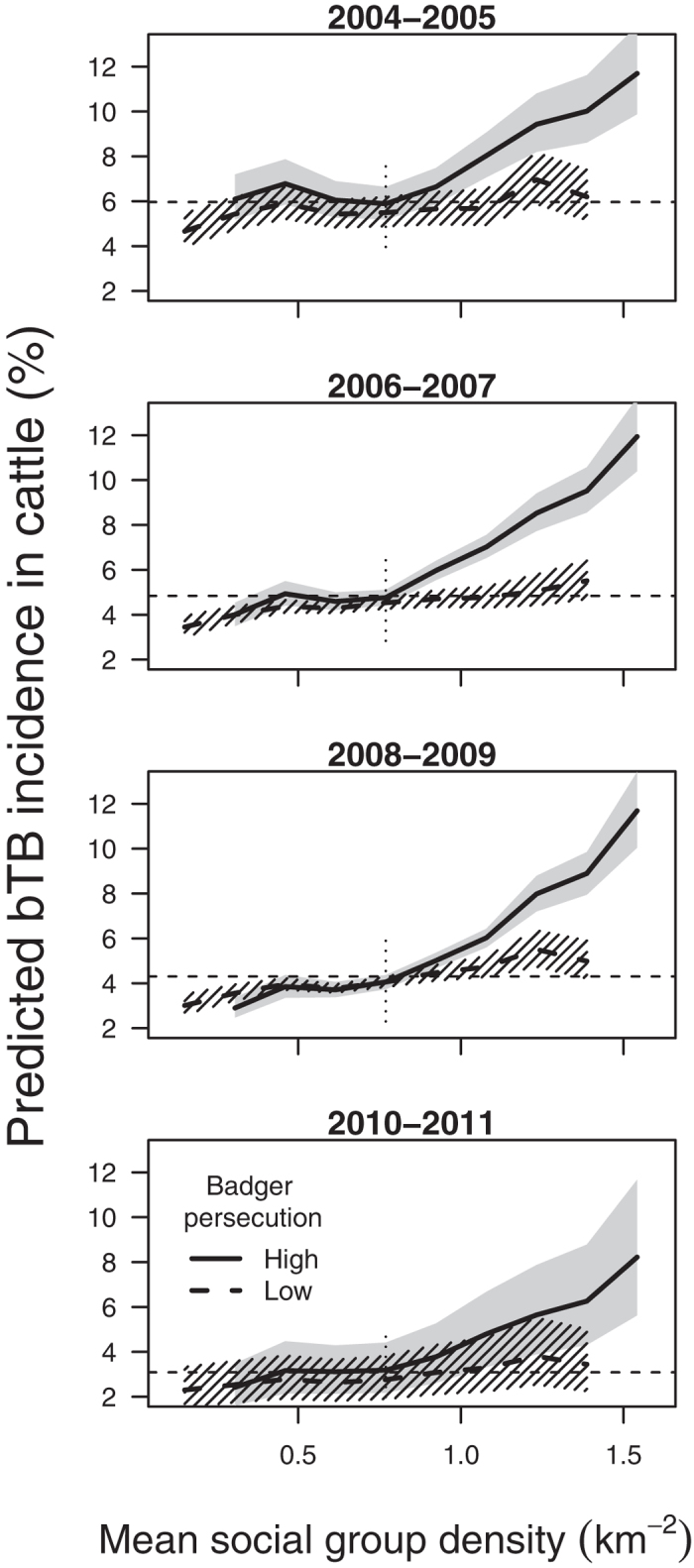
Predicted mean bTB risk across cattle herds in areas with high and low rates of badger persecution against badger social group (sett) density, Northern Ireland, 2004–2011. Herds were classified by social group density (10 bins) and mean risk calculated for each bin. High persecution areas had a probability of sett disturbance >0.35. Dashed horizontal lines indicate overall mean predicted bTB incidence for each time period (across all areas). Vertical dotted lines indicate the upper quartile of sett density, 0.83 groups km^−2^, separating low and high density areas. 95% confidence bands estimated by simulation from the fitted models. Details of models on which predictions are based are given in supplementary Table S4. Plot shows minimal changes in patterns of cattle bTB risk in areas with high and low rates of badger persecution over time.

**Figure 2:**
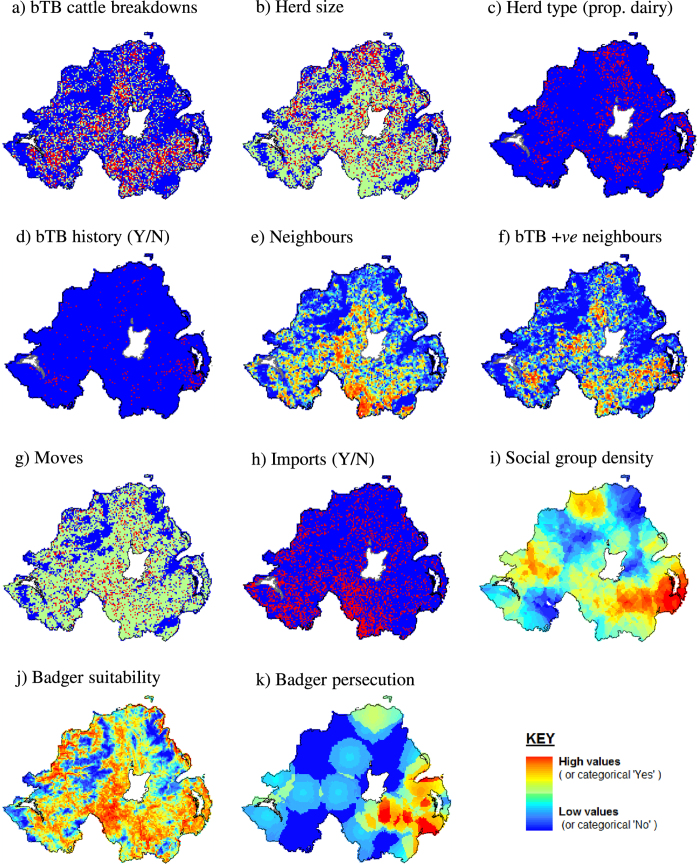
The geographical distribution of cattle bTB breakdowns and risk factors, Northern Ireland. Cattle variables measured 2004–2011, badger variables measured 2007–2008. (**a**) bTB cattle breakdowns, (**b**) herd size, (**c**) herd type [proportion of herds dairy], (**d**) bTB history [confirmed breakdown during past two years], (**e**) neighbours, (**f**) bTB +*ve* neighbours, (**g**) moves, (**h**) imports, (**i**) social group density, (**j**) badger suitability and (**k**) badger persecution. Maps created in ArcGIS v10.2 (ESRI, California, USA).

Of the badger variables, social group density, habitat suitability and persecution were retained in the final model along with interactions between them (Table 2). At mean levels of persecution and social group density, a two standard deviation increase in the suitability of the habitat for main sett construction was associated with a 12% increase in risk of a farm experiencing a breakdown (Table 2, for summary of variable distributions see Table 1). Breakdown risk was associated with variation in probability of badger persecution (sett disturbance) with an interaction between the effects of persecution and badger social group density (i.e. number of main setts). Areas with low rates of persecution sustained small increases in bTB risk with increasing social group density but in areas with high rates of persecution, bTB risk increased considerably with social group density across all time periods (Table 2, Fig. 1). In high persecution areas the relationship was non-linear with increases in predicted incidence only in areas with >0.83 social groups per km^2^. There was a positive correlation between badger social group density and persecution (ρ = 0.58, P < 0.001). Areas with the highest social group density and highest rates of persecution were in the south-east of the region i.e. Co. Down (Fig. 2). Estimates of associations between predictor variables (both badger and cattle) and bTB risk using only data from grid squares in which badgers were surveyed were consistent with those obtained using the full dataset (see sensitivity analysis, Supplementary materials).

### Temporal trends in bTB risk

The majority of grid squares had low badger social group density (interpolated) and a low rate of persecution and so the predicted bTB incidence in areas of low persecution tracked the overall decreasing trend in cattle bTB incidence better than the high persecution profile (Fig. 1). Areas with high badger social group densities (in the upper quartile; >0.83 groups km^−2^) and high rates of persecution remained at elevated risk of breakdown (bTB hotspots) regardless of temporal trends in bTB incidence in the rest of the country. In low persecution areas the weak positive association between social group density and incidence was similar before (2004–2007) and after (2008–2011) collection of badger population data. Breakdown risks before the badger survey were greater in high compared with low persecution areas, especially at higher badger social group densities (Fig. 1). High persecution areas remained at greater risk than low persecution areas after the badger survey and the decreases in risk during these periods (2008–2009 and 2010–2011) were similar across both persecution categories. There was no apparent change in the slope of the risk profile of high persecution areas moving from the period immediately before (2006–2007) to after (2008–2009) the badger survey or convergence of the high and low persecution risk profiles after the badger survey.

## Discussion

In our population scale comparison of bTB risk factors for new herd breakdowns, cattle-related factors were a far better predictor of bTB risk than badger-related factors in terms of model fit. Herd size was a key predictor and high bTB risks have been previously associated with large herds in the UK and Ireland^10^,^11^,^29^,^30^. Similarly, bTB breakdowns on neighbouring farms, high local prevalence or a history of bTB have been strongly associated with increased risk of breakdown^30^,^31^,^32^,^33^. Recurrent breakdowns may result either from re-infection, potentially from the same source if biosecurity practices remain unchanged, or from infection persisting undetected within a herd^34^,^35^. The moderate sensitivity of the tuberculin skin test, the primary diagnostic test used to screen herds for bTB, has been highlighted^36^,^37^,^38^ and so the contribution of undetected infection towards recurrent breakdowns may be considerable^35^,^38^,^39^,^40^. In contrast with a recent study which reported that dairy herds in Northern Ireland were likely to have breakdowns at more frequent intervals than beef herds^33^, we found no additional risk associated with dairy herds, possibly because different methods were used to identify dairy herds (breed vs. milk licence based). Buying multiple batches of cattle is an established bTB risk factor^40^,^41^, especially when animals are sourced from herds with a history of bTB^42^,^43^ and this was associated with the second largest increase in bTB risk in our study (after herd size). There was an independent additive effect of international imports from the Republic of Ireland on bTB risk despite mandatory skin testing of all cattle prior to importation, further illustrating the limitations of current diagnostic tests in preventing movement of infected cattle. These results indicate that there remains considerable scope for reducing bTB risk through controls focused solely on addressing the well-established risks of cattle movement and persistent infection, by maintaining truly closed herds, enhancing biosecurity where possible and by efforts to improve consistency and sensitivity of the tuberculin test^44^ or to employ supplementary diagnostic tests more effectively to detect persistent infection^45^. As there were no major changes in bTB control policy and associations between bTB incidence and cattle population variables remained consistent throughout the study period, the decreasing trend in incidence is unlikely to be due to changes in the bTB control programme and remains largely unexplained.

Variation in incidence of bTB in cattle associated with the badger population was small in comparison with cattle variables but high badger social group density, habitat suitability and badger persecution were associated with elevated cattle bTB risk. The highest risks were found in areas of high badger social group density coupled with high rates of persecution through sett interference, with two potentially non-exclusive explanations for such an association. Firstly, badger persecution may be initiated in response to high local cattle bTB prevalence in an effort to protect herds from (re)infection, especially where badgers are common and, hence, a more visible perceived threat and accessible target^20^. Secondly, high persecution may lead to disruption of badger social groups and subsequent migration and transfer of the disease to proximate cattle herds, increasing bTB risk. This mechanism, the so called ‘perturbation effect’ has been observed in response to both large and small scale badger culling trials in Great Britain^46^, bolstering arguments against badger culling as a means to reduce bTB risk^18^. Our analysis of bTB risk profiles before and after the badger data were collected provides some indication of the relative influence of the two mechanisms. Badger persecution was more common in areas that had a history of high cattle bTB risk, indicating that responsive persecution is taking place in areas where badgers are perceived to be a threat. We found no evidence that badger persecution reduced bTB risk; risk profiles in both high and low persecution areas were very similar immediately before and after the badger survey. Our results do not exclude the possibility that bTB risk differentials among areas are maintained by continued high levels of persecution, potentially through badger population perturbation. The two processes, responsive persecution and perturbation may operate in parallel, leading to positive feedbacks which may contribute to the persistence of bTB hotspots in certain areas independent of established cattle risk factors. Similarly, we cannot rule out an inhibitory effect of high badger persecution on cattle bTB incidence (i.e. without persecution, incidence might have been higher still) or that patterns of persecution may have changed following the badger survey, potentially obscuring the true relationship between persecution and bTB risk. The effect of badger culling on bTB risk has been extensively studied and led to the formulation of the perturbation hypothesis^17^. Our findings indicate that badger sett disturbance (with or without killing of the occupants) is also associated with high cattle bTB risk but that the association may be responsive as well as causal. Without repeated surveys of badger density and persecution linked to cattle bTB incidence data in high and low persecution areas, it is difficult to speculate which is the dominant process. To investigate whether persecution does lead to perturbation, badger movement patterns in high and low persecution areas could be compared using GPS-telemetry or molecular epidemiology methods^47^,^48^.

An important consideration is whether these findings, based on data from Northern Ireland can be extrapolated to other parts of the British Isles. The relative influence of badgers on bTB dynamics in Great Britain and the Republic of Ireland has been widely debated, with large scale badger culling trials in each country yielding dramatically different results and policy recommendations^7^. Proactive culling of badgers in the Irish ‘Four Areas’ trial was considered effective in reducing cattle bTB incidence, with dramatic reductions in cull areas in comparison with reference areas^14^. In Great Britain, evidence in favour of culling was more equivocal; the Randomized Badger Culling Trial (RBCT) in Great Britain revealed only modest decreases in cattle bTB incidence in cull areas but increases of a similar magnitude in surrounding areas^46^. Explanations for these differences include inter-country variation in study design, bTB testing and control programmes and cattle herd demographics (average herd size is greater in Great Britain) but also badger population structure^7^.

Badger population density in Ireland (in both Northern Ireland and the Republic of Ireland) is thought to be low, having remained relatively stable whilst the population in Great Britain has increased^12^,^22^,^49^. Therefore, considering solely badger population density, the association between high persecution and elevated bTB incidence that we observed at higher social group densities might also be expected to occur in Great Britain. Incidence of persecution has not been surveyed in Great Britain in recent years but historical trends indicate it is likely to be lower than in Northern Ireland^12^,^20^ and so areas with sufficient persecution to show this association might be limited. Furthermore, the extent to which badger social group behaviour varies between Northern Ireland and Great Britain, particularly with respect to ranging and inter-group contact remains largely unknown. For example, a study in Great Britain found that bTB positive badgers were likely to range further and have more contact with other social groups than bTB negative badgers^48^ but similar tracking studies have only recently been initiated in Northern Ireland. Given these considerations, care should be taken when extrapolating our findings to Great Britain but given the greater similarities in both badger and cattle populations we suggest that our results are more representative of the situation in the Republic of Ireland.

Despite the population-scale dataset used, our models had relatively modest predictive power and considerable unexplained variation, indicating that the selected variables did not capture all of the dynamics of the system and that, in common with many observational studies, unquantified covariates were likely to have had an influence on outcomes. Most of the herd-level predictors identified in a global review of bTB risk factors^2^ were included but there was considerable residual variation among cattle in risk. Age, sex, breed, concurrent infection(s) and genetic susceptibility to bTB can influence the probabilities of infection and skin test detection, determining whether, and at what point, a herd breakdown may be detected^2^,^50^,^51^,^52^. Considering bTB risk at the individual-level rather than at the herd-level is likely to refine risk estimates and might highlight particular high-risk groups of cattle (perhaps older dairy cows and bulls) that should be monitored more closely in disease surveillance programmes. Also, our measures of badger population variables, whilst giving comprehensive coverage of the study region were collected at a coarser scale than the available cattle population information and so spatial kriging and species distribution models were used to interpolate badger variables for each cattle herd location^22^. Additional uncertainty associated with spatially interpolated values is likely to have contributed to the modest explanatory power of our model but is unlikely to have substantially altered our findings as estimates obtained using only data from surveyed squares were consistent with those from the full dataset. Our findings have implications for the formation of policies to reduce bTB risk in the cattle population. We have shown that established cattle risk factors are more closely associated with bTB risk than features of the badger population, highlighting the importance of preventing transmission within the primary population through discouraging unnecessary cattle movement and increasing further the efficacy of testing programmes. Interventions to address these issues, including risk-based trading and bTB testing programmes^10^,^53^ are likely to be considerably less expensive and more publicly acceptable than schemes based on culling of badgers and may be more cost-effective and easier to monitor. However, there is evidence that transmission of *M. bovis* between the two host populations occurs relatively frequently^54^ and the associations between bTB risk, badger social group density and persecution that we observed indicate that interventions to control bTB in badgers may also play a role, especially where badger-cattle transmission acts to seed new breakdowns^55^. In particular, this is the first study to highlight the potential importance of badger population disturbance other than officially sanctioned culling in sustaining the bTB epidemic. Persecution did not appear to substantially reduce cattle bTB risk and may have exacerbated the problem by triggering perturbation. Therefore, it may be beneficial to inform stakeholders of the risks incurred by disturbing setts. These findings should also be considered when designing bTB control programmes that use sub-lethal interventions in the badger population (including proposed badger vaccination programmes^56^) and efforts should be made to minimise disturbance of badger social group structure in the implementation of such programmes.

## Additional Information

**How to cite this article**: Wright, D. M. *et al.* Herd-level bovine tuberculosis risk factors: assessing the role of low-level badger population disturbance. *Sci. Rep.* **5**, 13062; doi: 10.1038/srep13062 (2015).

## Supplementary Material

Supplementary Information

## Figures and Tables

**Table 1:** List of candidate explanatory variables predicting cattle bTB breakdown using logistic regression.

**Variable**	**Description**
(a) Cattle risk factors	
Herd size	Number of animals present on 1^st^ January categorised as 0–10, 10–100 and >100 cattle.
Herd type	Beef or Dairy (if >50% of the herd dairy breeds).
bTB history	Whether the herd had a history of (confirmed) bTB. The number of years since a herd had an open breakdown (7 categories: No history, 1, 2, 3, 4, 5, 6–10; i.e. 1 = breakdown in the previous year). The baseline group was herds with no bTB history during the past 10 years. Second breakdowns in the same calendar year were excluded from the risk set.
Neighbours	Number of active neighbouring herds in the previous calendar year (range 0–36, mean 8.75, SD = 4.41). Neighbours defined as herds within a 1 km radius of the focal farm centroid.
bTB +*ve* neighbours	Number of neighbouring herds in which a confirmed bTB breakdown occurred during the previous year (range 0–11, mean = 0.44, SD = 0.77).
Moves	Number of batches of animals moved into the herd during the previous year (0, 1–10, >10).
Imports	Animals imported from the Republic of Ireland (ROI) during the previous year (yes/no)
Year	Year
(b) Badger risk factors (for full Methods see Reid *et al.* 2011)	
Social group density	Density of badger main setts per km^2^ interpolated using Spatial Kriging and extracted at the herd location (range 0.075–1.618, mean = 0.70, SD = 0.25).
Habitat suitability	Index for the digging of main setts derived from a spatially explicit Species Distribution Model (range 1.03–9.77, mean = 6.10, SD = 1.69).
Persecution	Index of badger persecution (range 0–1, mean = 0.26, SD = 0.25) indicating the probability of interference with sett structure during 2007/08 e.g. recent digging, blocking of entrances etc.

**Table 2:** Adjusted risk of new bTB breakdowns in cattle herds in Northern Ireland, 2004–2011.

**Variable**	**Unit**	**OR**	**CI**
(a) Cattle risk factors			
Herd size	0–10	0.23	(0.20, 0.25)***
	10–100	1.00	
	100+	2.27	(2.15, 2.39)***
Herd type	Beef	1.00	
	Dairy	1.00	(0.95, 1.06)
bTB history	No history	1.00	
	1	2.33	(2.17, 2.49)***
	2	2.10	(1.94, 2.26)***
	3	1.83	(1.68, 1.99)***
	4	1.83	(1.67, 2.01)***
	5	1.69	(1.53, 1.88)***
	6–10	1.44	(1.33, 1.57)***
Neighbours		0.99	(0.99, 1.00)*
bTB +*ve* neighbours		1.26	(1.23, 1.29)***
Moves	0	1.00	
	1–10	1.29	(1.22, 1.38)***
	10+	2.05	(1.90, 2.21)***
Imports	No	1.00	
	Yes	1.17	(1.07, 1.28)***
(b) Badger risk factors			
Social group density		1.08	(1.02, 1.15)**
Habitat suitability		1.12	(1.07, 1.18)***
Persecution		1.06	(1.00, 1.12).
Habitat suitability * Persecution		1.15	(1.01, 1.30)*
Social group density * Persecution		1.39	(1.29, 1.49)***
Social group density * Suitability		0.79	(0.70, 0.89)***

Estimated odds ratios (ORs) and 95% Confidence Intervals (CIs) for a range of cattle- and standardized badger-related risk factors given. Ranges for standardized badger population variables: social group density −1.24 to +1.84; habitat suitability −1.51 to + 1.09; persecution −0.51 to +1.49. Significance values: ***P < 0.001; **P < 0.001; *P < 0.05; *P* < 0.1.
