# Supplementary material for: Herd-level bovine tuberculosis risk factors: assessing the role of low-level badger population disturbance
Source: Sci Rep. 2015 Aug 17;5:13062. doi: 10.1038/srep13062 (PMC4642523; doi:10.1038/srep13062)

# Herd-level bovine tuberculosis risk factors: assessing the role of low-level badger population disturbance

David M. Wright^1,2^*, Neil Reid^1,3,4^, W. Ian Montgomery^1,3,4^, Adrian R. Allen^5^, Robin A. Skuce^1^,^5^ & Rowland R. Kao^2*^

^1^ School of Biological Sciences, Queen’s University Belfast, Belfast, BT9 7BL, Northern Ireland.

^2^ College of Medical, Veterinary and Life Sciences, University of Glasgow, Glasgow, G61 1QH, Scotland.

^3^ *Quercus*, School of Biological Sciences, Queen’s University Belfast, Belfast, BT9 7BL, Northern Ireland (UK).

^4^ Institute for Global Food Security (IGFS), Queen’s University Belfast, Belfast, BT9 5BN, Northern Ireland (UK).

^5^ Veterinary Sciences Division, Agri-Food and Biosciences Institute, Stormont, Belfast BT4 3SD, Northern Ireland.

* Joint corresponding authors: [david.m.wright@cantab.net](mailto:david.m.wright@cantab.net); Rowland.Kao@glasgow.ac.uk

# Supplementary Material

*Sensitivity analyses*

A sensitivity analysis was conducted by refitting the best-fitting models using only data from herds located in grid squares in which badger setts were surveyed (313 herds, 212 grid squares). These results were compared with those obtained from the full dataset to determine whether interpolated values for badger variables were driving the patterns observed using the full dataset. Estimates of associations between predictor variables (both badger and cattle) and bTB risk using the reduced dataset were consistent with those obtained using the full dataset (compare Table 2 with Table S5). All associations were in the same direction with the exception of the herd type indicator (1.00 using full dataset and 0.81 using reduced dataset) and all point estimates from the full model were within the 95%CIs for the reduced model with the exception of the estimate for the social group density * habitat suitability interaction. For the majority of variables the opposite was not true (i.e. point estimates from the reduced model were outside the CIs for the full model) and estimates from the reduced model were of greater magnitude than those from the full model. Confidence intervals for the reduced model were wider than for the full model, due to the considerably reduced sample size.

*Functional forms*

Some of the predictor variables had a skewed distribution (in particular badger persecution risk) and so we assessed whether different functional forms of the relationships with bTB risk would be more appropriate and improve model fit, by transforming the predictor variables (square root transformation, classification into low and high persecution areas). Although a square root transformation of the habitat suitability variable marginally improved overall model fit (model 10, Table S3), we selected model 7 as the preferred badger-cattle model because the relative influences of the different badger variables are more easily interpretable if kept on a common linear scale.

*Model fit*

The area under the ROC curve (AUC) gives an indication of a model’s predictive power and overall goodness-of-fit. Values may range from 0.5 (no discrimination) to 1 (perfect classification of outcomes). The selected overall model (Table 2, Table S3 – model 7) had an AUC of 0.757, an acceptable value but by no means outstanding, indicating that much remains unexplained by the model regarding the transmission dynamics of bTB.

The model containing only badger variables (model 0) had a much higher predictive power (AUC = 0.928) than both the cattle only (model 1) and cattle-badger (model 7) models (AUC = 0.759 and 0.763 respectively), despite being a much poorer quality model (model 0 had the highest AIC value, Table S3). This discrepancy was found to be related to the herd-level random effects specified in all models. In both the cattle only and combined cattle-badger models, a large proportion of the herd-level variation was associated with the cattle variables and so residual herd-level variation was low (i.e. random herd effects were small, SD < 0.0001). In the badger only model all of the herd-level variation was associated with the herd random effects, with a correspondingly large variation in herd effects (SD = 1.26). The badger model therefore had much greater flexibility to model variation among herds, effectively allowing an unrestricted estimate of risk for each herd, and so had greater predictive power at the expense of parsimony and overall model quality (an example of over-fitting). When the herd-level random effect was removed, AUC of the three models showed the expected pattern based on AIC (AUC: cattle only = 0.759; badger only = 0.604; combined = 0.763).

**Table S1** Number of active cattle herds and new bTB breakdowns in Northern Ireland from 2004 to 2011. The numbers of herds excluded from the incidence calculations, having an existing breakdown which extended into that calendar year are also given.

| **Year** | **Herds** | **Breakdowns** | **Incidence (%)** | **Excluded** |
| --- | --- | --- | --- | --- |
| 2004 | 24,538 | 1,557 | 6.3 | 2728 |
| 2005 | 24,707 | 1,376 | 5.7 | 2205 |
| 2006 | 24,758 | 1,233 | 5.2 | 1772 |
| 2007 | 24,649 | 1,149 | 4.6 | 1454 |
| 2008 | 24,209 | 1,039 | 4.2 | 1471 |
| 2009 | 23,879 | 1,024 | 4.2 | 1394 |
| 2010 | 23,828 | 921 | 3.9 | 1279 |
| 2011 | 23,895 | 565 | 2.4 | 1017 |
| **Total** | **194,463** | **8,864** | **4.6** | **13,320** |

Note that these estimates differ from official estimates of herd-level incidence^1^ because definitions of active herds and risk sets for new breakdowns differ between studies, along with estimation methods. Overall trends in incidence are consistent across studies.

1. Tuberculosis disease statistics in Northern Ireland – December 2012

<http://www.dardni.gov.uk/index/statistics/animal-disease-statistics/statistics-tuberculosis.htm>

(accessed October 2014).

**Table S2** Characteristics of non-TB and TB herds, Northern Ireland, 2004-2011. Percentage of farm years at risk are given for categorical variables. Mean and SD given for continuous variables.

| **Variable** | **Unit** | **Non-TB** | **TB** |
| --- | --- | --- | --- |
|  |  |  |  |
| **a) Cattle risk factors** |  |  |  |
| Herd size | 0-10 | 24.8 | 3.8 |
|  | 10-100 | 59.3 | 52.9 |
|  | 100+ | 15.9 | 43.3 |
| Herd type | Beef | 83.3 | 73.9 |
|  | Dairy | 16.7 | 26.1 |
| bTB history | No history | 69.8 | 43.4 |
|  | 1 | 5.4 | 16.2 |
|  | 2 | 4.8 | 11.5 |
|  | 3 | 4.3 | 8.1 |
|  | 4 | 3.9 | 6.9 |
|  | 5 | 3.5 | 5.3 |
|  | 6-10 | 8.4 | 8.6 |
| Neighbours |  | 8.76 (4.42) | 8.60 (0.97) |
| bTB *+ve* neighbours |  | 0.43 (0.76) | 0.65 (0.97) |
| Moves | 0 | 31.6 | 15.5 |
|  | 1-10 | 57.8 | 59.1 |
|  | 10+ | 10.6 | 25.3 |
| Imports | No | 96.3 | 92.8 |
|  | Yes | 3.7 | 7.2 |
|  |  |  |  |
| **b) Badger risk factors** |  |  |  |
| Social group density |  | 0.70 (0.25) | 0.75 (0.29) |
| Habitat suitability |  | 6.09 (1.69) | 6.37 (1.53) |
| Persecution |  | 0.25 (0.25) | 0.30 (0.28) |

**Table S3** Comparison of models of new TB breakdowns in Northern Ireland from 2003-2011. Models ordered by AIC values.

| **Model** | **Description** | **AIC** |
| --- | --- | --- |
| 10 | cattle + social group density * habitat suitability (square root transformation) * badger persecution | 64105.83 |
| 7 | cattle + social group density * habitat suitability * badger persecution **(cattle-badger model)** | 64106.17 |
| 9 | cattle + social group density * habitat suitability * badger persecution (square root transformation) | 64113.79 |
| 8 | cattle + social group density * habitat suitability * badger persecution (high-low classification) | 64115.37 |
| 11 | cattle + social group density (square root transformation) * habitat suitability * badger persecution | 64115.97 |
| 6 | cattle + habitat suitability + social group density * badger persecution | 64117.85 |
| 5 | cattle + social group density + habitat suitability + badger persecution | 64197.83 |
| 2 | cattle + social group density | 64252.63 |
| 4 | cattle + badger persecution | 64260.63 |
| 3 | cattle + habitat suitability | 64364.24 |
| 1 | cattle variables only **(base cattle model)** | 64405.49 |
| 0 | badger variables only **(badger model)** | 69841.75 |

**Table S4.** Adjusted risk of new bTB breakdowns in cattle herds in Northern Ireland, 2004-2011. Estimated odds ratios (ORs) and 95% Confidence Intervals (CIs) for a range of cattle- and standardized badger-related risk factors given. Ranges for standardized badger population variables: social group density -1.24, 1.84; habitat suitability -1.51, 1.09; persecution -0.51, 1.49. Badger survey conducted 2007-2008.

|  |  | **2004-2005** | | **2006-2007** | | **2008-2009** | | **2010-2011** | | |
| --- | --- | --- | --- | --- | --- | --- | --- | --- | --- | --- |
| **Variable** | **Unit** | **OR** | **CI** | **OR** | **CI** | **OR** | **CI** | **OR** | **CI** |  |
| **a) Cattle risk factors** |  |  |  |  |  |  |  |  |  |  |
| Herd size | 0-10 | 0.19 | (0.15,0.23) | 0.25 | (0.20,0.31) | 0.28 | (0.22,0.35) | 0.20 | (0.15,0.27) |  |
|  | 10-100 | 1 |  | 1 |  | 1 |  | 1 |  |  |
|  | 100+ | 2.32 | (2.11,2.55) | 2.31 | (2.08,2.57) | 2.30 | (2.06,2.57) | 2.03 | (1.78,2.31) |  |
| Herd type | Beef | 1 |  | 1 |  | 1 |  | 1 |  |  |
|  | Dairy | 0.94 | (0.85,1.04) | 0.90 | (0.81,1.01) | 1.14 | (1.02,1.29) | 1.13 | (0.99,1.29) |  |
| bTB history | No history | 1 |  | 1 |  | 1 |  | 1 |  |  |
|  | 1 | 2.34 | (2.10,2.61) | 2.37 | (2.07,2.70) | 2.27 | (1.94,2.66) | 2.36 | (1.97,2.83) |  |
|  | 2 | 2.02 | (1.77,2.29) | 2.16 | (1.87,2.48) | 2.21 | (1.88,2.61) | 2.04 | (1.67,2.50) |  |
|  | 3 | 1.58 | (1.34,1.87) | 2.10 | (1.81,2.44) | 1.72 | (1.44,2.05) | 1.94 | (1.56, 2.41) |  |
|  | 4 | 1.80 | (1.50,2.16) | 1.68 | (1.41,2.01) | 2.00 | (1.69,2.37) | 1.87 | (1.50,2.34) |  |
|  | 5 | 1.61 | (1.31,1.98) | 1.82 | (1.48,2.23) | 1.84 | (1.53,2.21) | 1.47 | (1.16,1.87) |  |
|  | 6-10 | 1.16 | (0.80,1.67) | 1.29 | (1.08,1.56) | 1.49 | (1.29,1.72) | 1.55 | (1.34,1.80) |  |
| Neighbours |  | 0.94 | (0.85,1.04) | 1.01 | (1.00,1.02) | 0.99 | (0.98,1.00) | 0.98 | (0.97,0.99) |  |
| bTB *+ve* neighbours |  | 1.23 | (1.18,1.28) | 1.29 | (1.23,1.35) | 1.27 | (1.20,1.34) | 1.25 | (1.16,1.34) |  |
| Moves | 0 | 1 |  | 1 |  | 1 |  | 1 |  |  |
|  | 1-10 | 1.12 | (1.01,1.25) | 1.40 | (1.24,1.58) | 1.48 | (1.30,1.69) | 1.23 | (1.06,1.44) |  |
|  | 10+ | 1.48 | (1.29,1.69) | 2.35 | (2.03,2.72) | 2.49 | (2.12,2.91) | 2.28 | (1.91,2.74) |  |
| Imports | No | 1 |  | 1 |  | 1 |  | 1 |  |  |
|  | Yes | 1.23 | (1.05,1.44) | 1.22 | (1.02,1.46) | 1.12 | (0.92,1.35) | 1.11 | (0.92,1.35) |  |
|  |  |  |  |  |  |  |  |  |  |  |
| **b) Badger risk factors** |  |  |  |  |  |  |  |  |  |  |
| Social group density |  | 0.99 | (0.89,1.09) | 1.11 | (0.99,1.24) | 1.22 | (1.08,1.37) | 1.08 | (0.94,1.24) |  |
| Habitat suitability |  | 1.18 | (1.08,1.28) | 1.06 | (0.96,1.17) | 1.11 | (1.01,1.24) | 1.12 | (0.99,1.26) |  |
| Persecution |  | 1.09 | (0.98,1.20) | 1.11 | (0.99,1.24) | 0.92 | (0.81,1.04) | 1.12 | (0.97,1.29) |  |
| Habitat suitability * Persecution |  | 1.18 | (0.95,1.47) | 1.05 | (0.82,1.34) | 1.32 | (1.01,1.72) | 1.09 | (0.80,1.47) |  |
| Social group density * Persecution |  | 1.30 | (1.15,1.48) | 1.35 | (1.18,1.55) | 1.47 | (1.28,1.70) | 1.44 | (1.22,1.70) |  |
| Social group density * suitability |  | 0.87 | (0.71,1.07) | 0.84 | (0.66,1.05) | 0.68 | (0.53,0.87) | 0.71 | (0.53,0.94) |  |

**Table S5.** Adjusted risk of new bTB breakdowns in cattle herds in 212 1 km^2^ grid squares surveyed for badger activity, Northern Ireland, 2004-2011.

Estimated odds ratios (ORs) and 95% Confidence Intervals (CIs) for a range of cattle- and standardized badger-related risk factors given. Ranges for standardized badger population variables: social group density -0.93, 1.34; habitat suitability -1.31, 0.96; persecution -0.51, 1.46. Badger survey conducted 2007-2008. Significance values: *** *P* < 0.001; ** *P* < 0.001; * *P* < 0.05; . *P* < 0.1.

|  |  |  | |
| --- | --- | --- | --- |
| **Variable** | **Unit** | **OR** | **CI** |
|  |  |  |  |
| **a) Cattle risk factors** |  |  |  |
| Herd size | 0-10 | 0.22 | (0.07,0.72)* |
|  | 10-100 | 1 |  |
|  | 100+ | 2.74 | (1.60,4.70)**** |
| Herd type | Beef | 1 |  |
|  | Dairy | 0.81 | (0.46,1.45) |
| bTB history | No history | 1 |  |
|  | 1 | 3.01 | (1.45,6.29)** |
|  | 2 | 2.21 | (1.02,4.80)* |
|  | 3 | 2.65 | (1.22,5.75)* |
|  | 4 | 3.21 | (1.47,7.03)** |
|  | 5 | 1.14 | (0.33,3.88) |
|  | 6-10 | 1.87 | (0.81,4.33) |
| Neighbours |  | 0.98 | (0.92,1.03) |
| bTB *+ve* neighbours |  | 1.22 | (0.95,1.57). |
| Moves | 0 | 1 |  |
|  | 1-10 | 1.79 | (0.92,3.50). |
|  | 10+ | 3.26 | (1.48,7.15)** |
| Imports | No | 1 |  |
|  | Yes | 1.25 | (0.51,3.09) |
|  |  |  |  |
| **b) Badger risk factors** |  |  |  |
| Social group density |  | 1.34 | (0.73,2.46) |
| Habitat suitability |  | 1.18 | (0.70,1.99) |
| Persecution |  | 0.89 | (0.49,1.59) |
| Habitat suitability * Persecution |  | 1.44 | (0.43,4.80) |
| Social group density * Persecution |  | 1.30 | (0.56,3.00) |
| Social group density * Suitability |  | 0.17 | (0.04,0.67)* |

**Supplementary Figure 1** Map of the location of 212 x 1km^2^ squares surveyed for badger setts in the Badger Survey of Northern Ireland 2007/08 (modified with permission from Reid *et al.,* 2008).

**
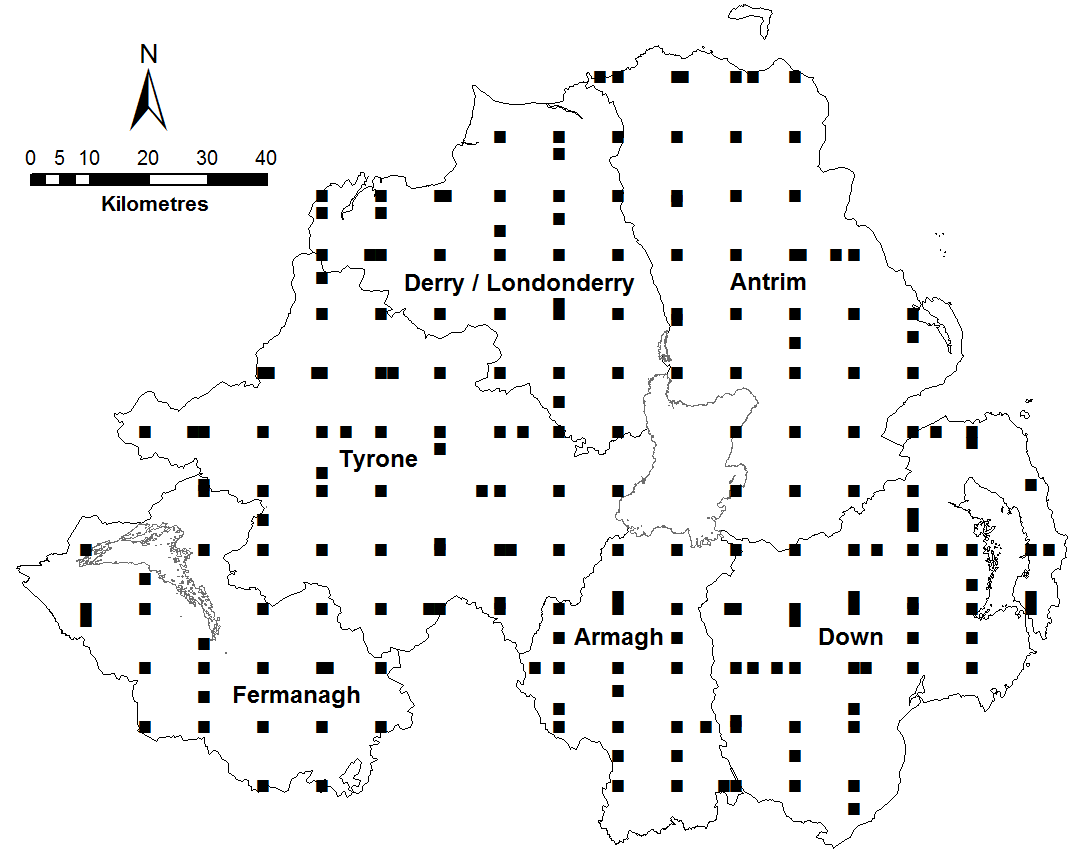
**

**Supplementary Figure 2** Variograms of the residual variance from combined cattle-badger model of bTB risk for each year of the study period. 2008 and 2009 lines overlap.


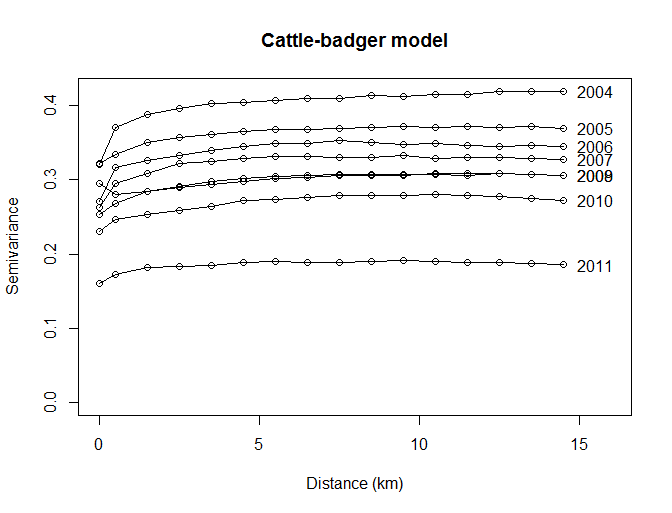

Supplement: Supplementary Information [file srep13062-s1.doc]
